# Supplementary material for: Cryo‐EM structure of a novel α‐synuclein filament subtype from multiple system atrophy
Source: FEBS Lett. 2024 Nov 7;599(1):33–40. doi: 10.1002/1873-3468.15048 (PMC11726156; doi:10.1002/1873-3468.15048)
Supplement: Supplementary file 1 — Fig. S1. Structures of MSA filaments reported by Schweighauser et al. Fig. S2. Flowchart of MSA filament data processing. Fig. S3. Model of MSA Type II2‐I2 mixed cofilament wherein adjacent Type II2 and Type I2 rungs were aligned using residues 57–93 of PF‐IIA. Fig. S4. Representative negative‐stain transmission electron micrographs of ex vivo MSA filaments. Fig. S5. Two‐dimensional class averages of MSA filaments extracted at two different box sizes. Fig. S6. Characterization of MSA Type II2 filaments in the dataset. Fig. S7. Characterization of MSA Type I2 filaments in the dataset. Table S1. Cryo‐EM data collection and model refinement statistics. Table S2. MSA filament type distribution across different brain regions in different cases. [file FEB2-599-33-s001.docx]

**SUPPORTING INFORMATION**

**Cryo-EM structure of a novel α-synuclein filament subtype from multiple system atrophy**

Nicholas L. Yan^1^, Francisco Candido^1^, Eric Tse^1,2^, Arthur A. Melo^1^, Stanley B. Prusiner^1,2,3^, Daniel A. Mordes^1,4,5^, Daniel R. Southworth^1,3^, Nick A. Paras^1,2^, and Gregory E. Merz*^1,2^

^1^ Institute for Neurodegenerative Diseases, Weill Institute for Neurosciences, University of California San Francisco, San Francisco, CA, USA

^2^ Department of Neurology, Weill Institute for Neurosciences, University of California San Francisco, San Francisco, CA, USA

^3^ Department of Biochemistry and Biophysics, University of California San Francisco, San Francisco, CA, USA

^4^ Department of Pathology, University of California San Francisco, San Francisco, CA, USA

^5^ Department of Pathology, Massachusetts General Hospital, Boston, MA, USA

*Correspondence: G. E. Merz, Department of Neurology, Institute for Neurodegenerative Diseases, University of California San Francisco, Sandler Neurosciences Center, 675 Nelson Rising Lane, San Francisco, CA 94158, USA

Tel: +1 415-502-7609

Email: Gregory.merz@ucsf.edu

**Table S1.** Cryo-EM data collection and model refinement statistics.

| **Data collection and processing** | **MSA Type II_2_** | **MSA Type I_2_** |
| --- | --- | --- |
| Microscope and camera | Titan Krios, K3 | |
| Magnification | 105,000 | |
| Voltage (kV) | 300 | |
| Electron exposure (e^−^/Å^2^) | 46 | |
| Dose rate (e^−^/physical pixel/sec) | 16 | |
| Exposure per frame (sec) | 0.024 | |
| Defocus range (µm) | -0.8 to -1.8 | |
| Physical pixel size (Å) | 0.834 | |
| Movies collected | 42,224 | |
| Box size (pixels) | 288 | |
| Interbox distance (Å) | 28 | |
| Initial segments extracted | 257,982 | |
| Final segments | 31,115 | 12,802 |
| Resolution (Å) | 3.2 | 3.3 |
| B-factor (Å^2^) | -73.0 | -74.8 |
| Helical rise (Å) | 4.76 | 4.76 |
| Helical twist (°) | -1.35 | -1.42 |
| **Refinement** | **MSA Type II_2_** | **MSA Type I_2_** |
| **Model composition** | | |
| Non-hydrogen atoms | 10,950 | 10,989 |
| Protein residues | 1,600 | 1,606 |
| Number of chains | 20 | 22 |
| **RSMD** | | |
| Bond lengths (Å) | 0.006 | 0.006 |
| Bond angles (°) | 0.586 | 0.530 |
| **Validation** | | |
| MolProbity score | 2.00 | 1.50 |
| Clashscore | 7.78 | 9.34 |
| Rotamer outliers (%) | 1.94 | 1.01 |
| Cβ outliers (%) | 0 | 0 |
| **Ramachandran plot** | | |
| Favored (%) | 94.87 | 98.59 |
| Allowed (%) | 5.13 | 1.41 |
| Outliers (%) | 0 | 0 |
| **PDB accession code** | **9CD9** | **9CDA** |
| **EMDB accession code** | **EMD-45464** | **EMD-45465** |

**Table S2**. MSA filament type distribution across different brain regions in different cases. Cases 1–5 were previously analyzed by Schweighauser et al. [[1]]. Case 6 was analyzed in this work.

| MSA Case | Brain region | Type I filaments (%) | Type II filaments (%) |
| --- | --- | --- | --- |
| 1 | Putamen | 80 | 20 |
|  | Cerebellum | ~0 | ~100 |
|  | Frontal cortex | ~100 | ~0 |
| 2 | Putamen | 20 | 80 |
|  | Frontal cortex | ~0 | ~100 |
| 3 | Putamen | ~100 | ~0 |
|  | Frontal cortex | ~0 | ~100 |
| 4 | Putamen | ~100 | ~0 |
| 5 | Putamen | ~0 | 100 |
|  | Frontal cortex | ~0 | 100 |
| 6 | Cerebellum | 7 | 93 |


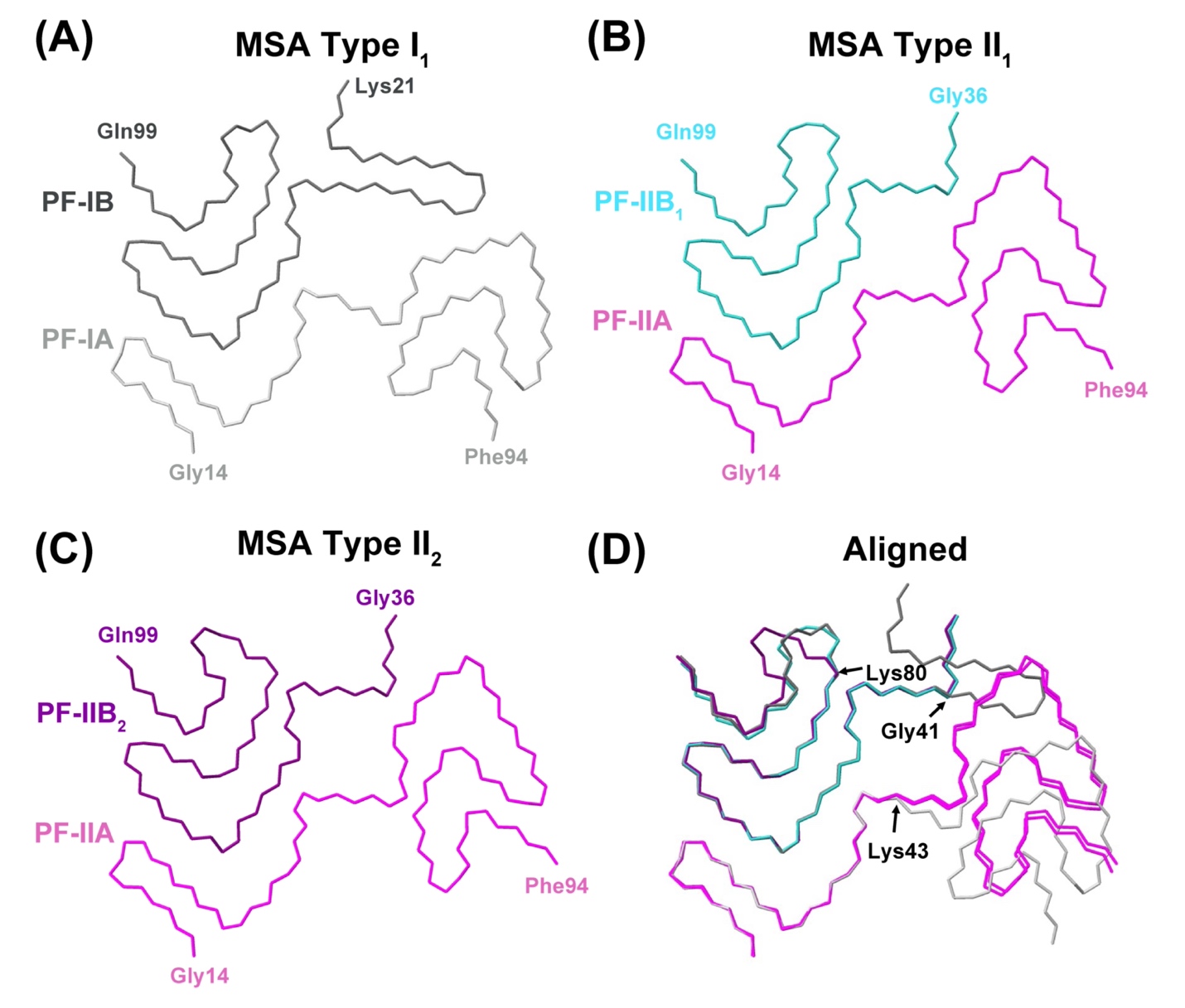


**Fig. S1** Structures of MSA filaments reported by Schweighauser et al. (A) Type I_1_ (PDB: 6XYO), (B) Type II_1_ (PDB: 6XYP), and (C) Type II_2_ (PDB: 6XYQ). (D) Alignment of the three filament conformations. In (A–C), residues at the termini of the ordered region of each protofilament are labeled. In (D), residues at the start or end of regions where the filament conformations diverge are labeled.


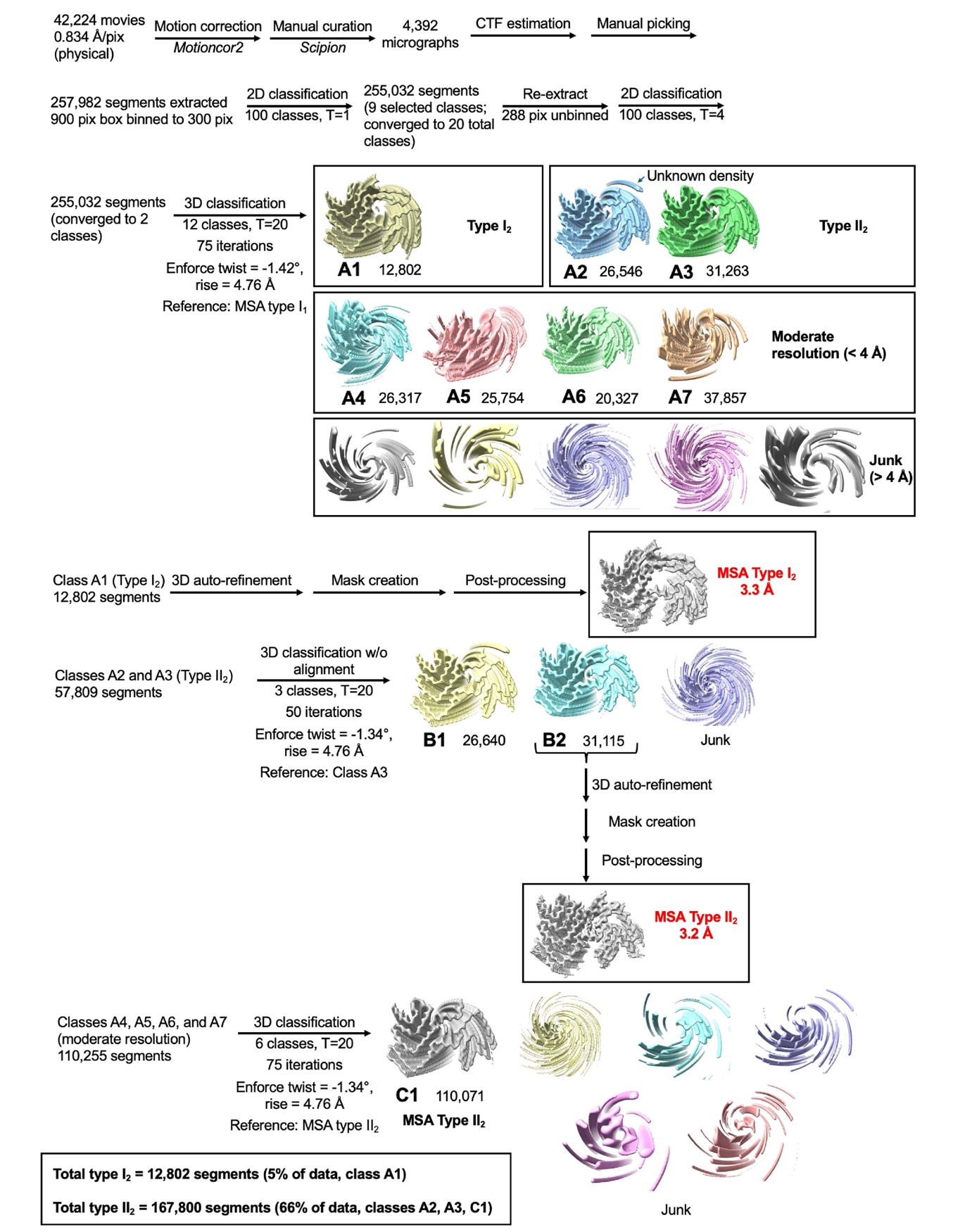


**Fig. S2** Flowchart of MSA filament data processing. Unless otherwise indicated, all data processing procedures were performed in RELION 4.


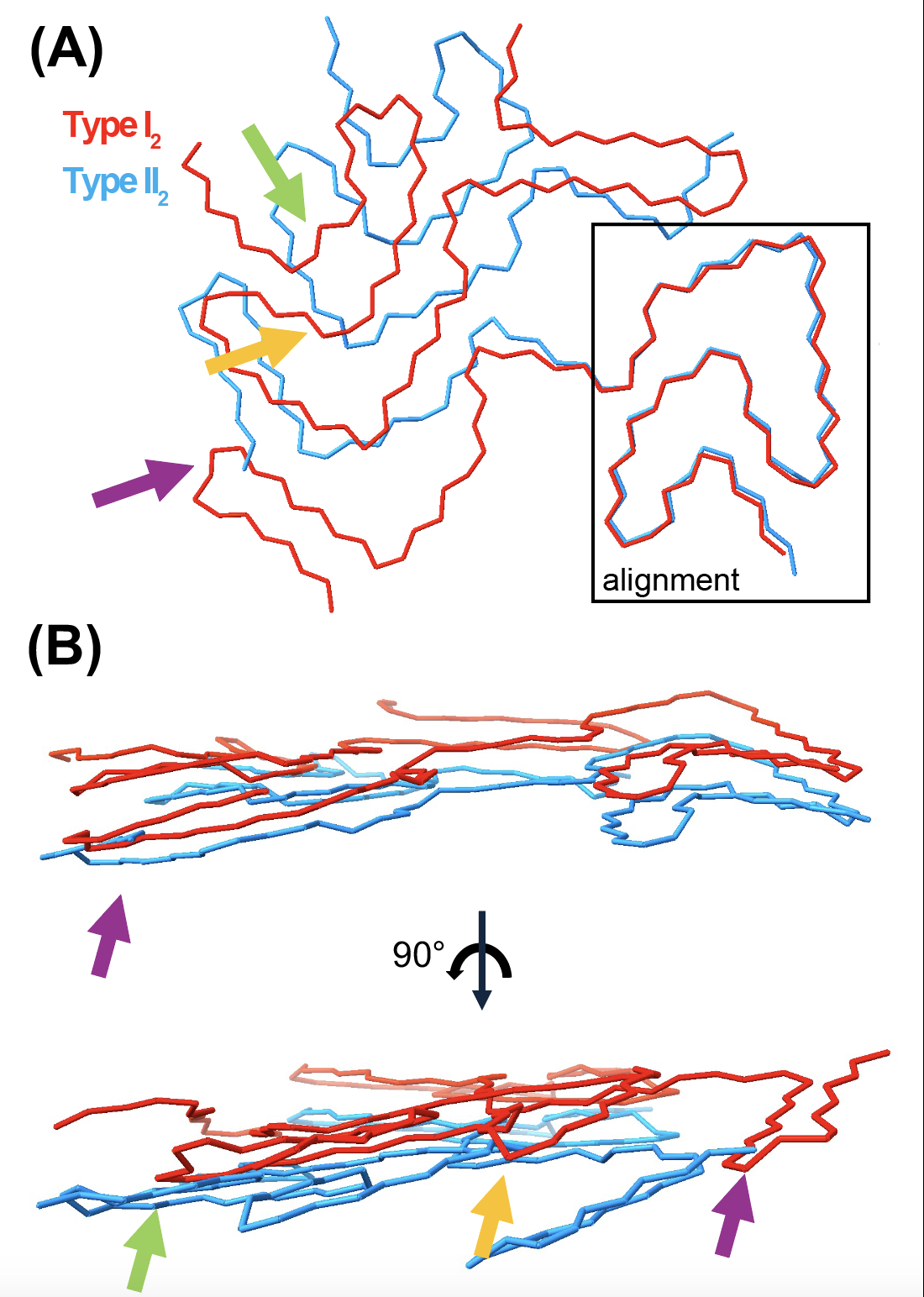


**Fig. S3** Model of MSA Type II_2_-I_2_ mixed cofilament wherein adjacent Type II_2_ and Type I_2_ rungs were aligned using residues 57–93 of PF-IIA (inset). (A) Cross-section of model. (B) Side-on views demonstrating severe steric clashes (arrows) between adjacent protofilaments.

**
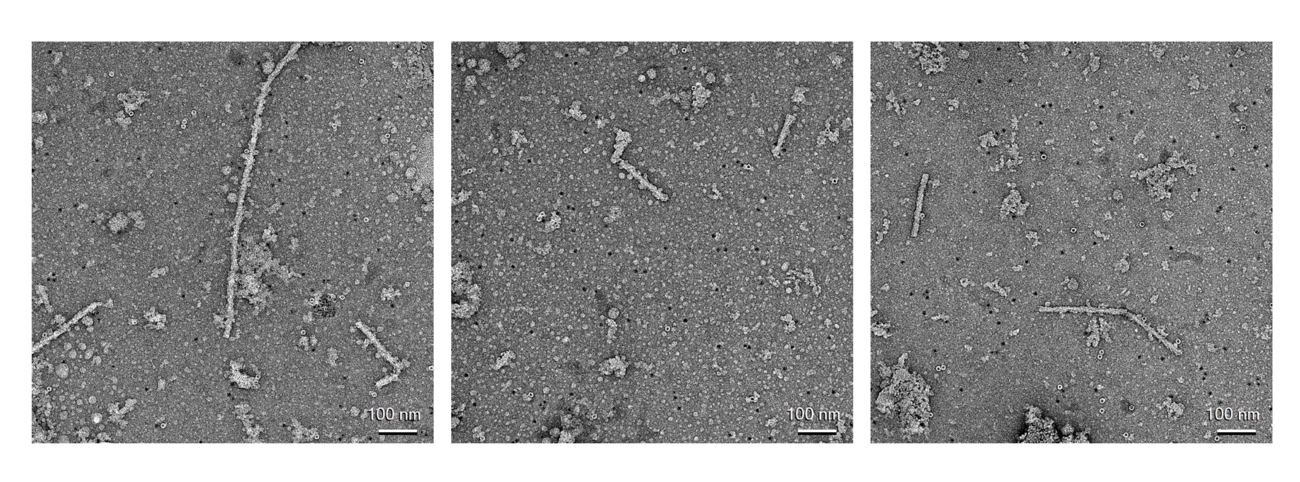
**

**Fig. S4** Representative negative-stain transmission electron micrographs of ex vivo MSA filaments.


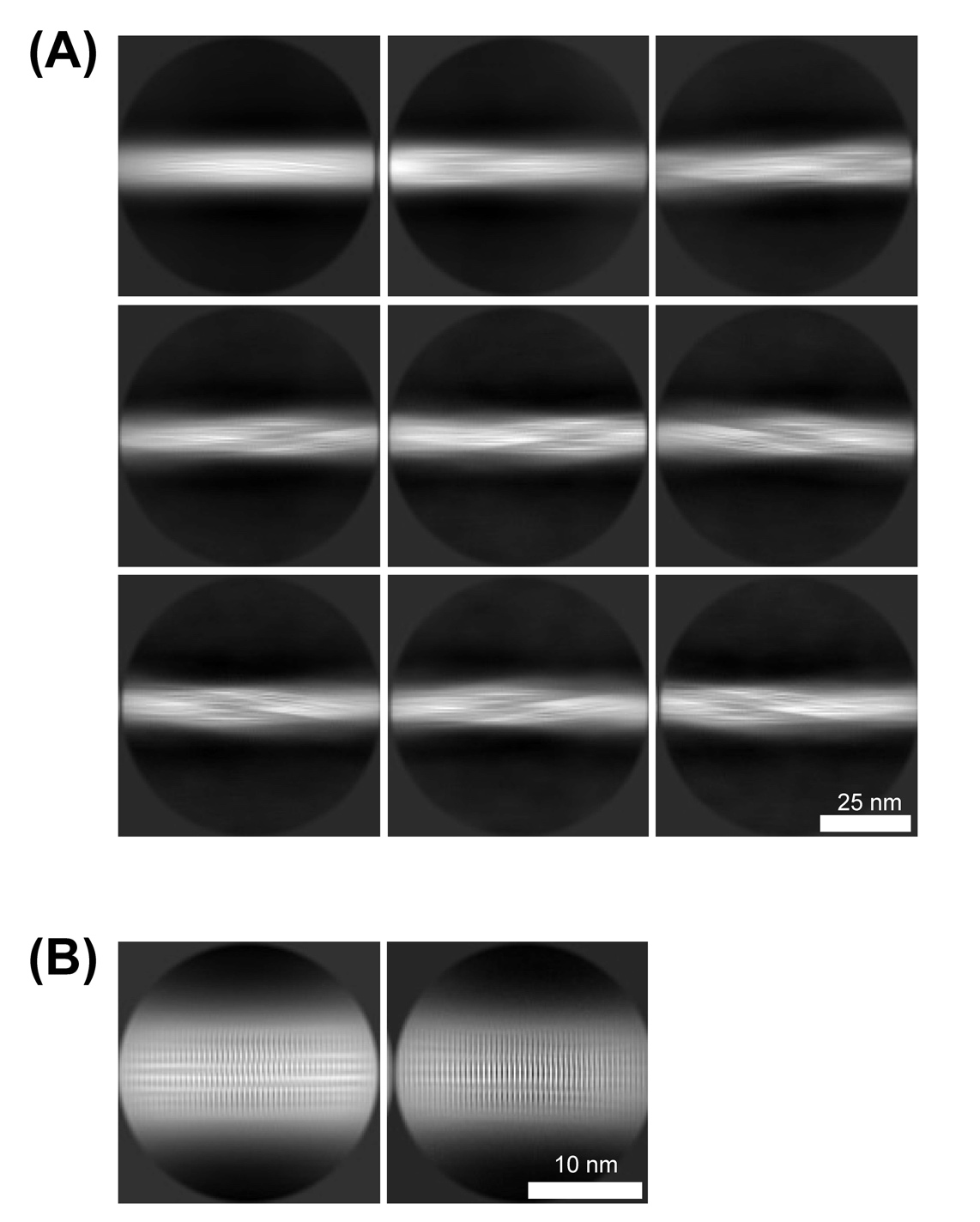


**Fig. S5** Two-dimensional class averages of MSA filaments extracted at two different box sizes. (A) At 900 pixels downscaled to 300 pixels. (B) At 288 pixels. Segments corresponding to these classes were used for 3D classification.


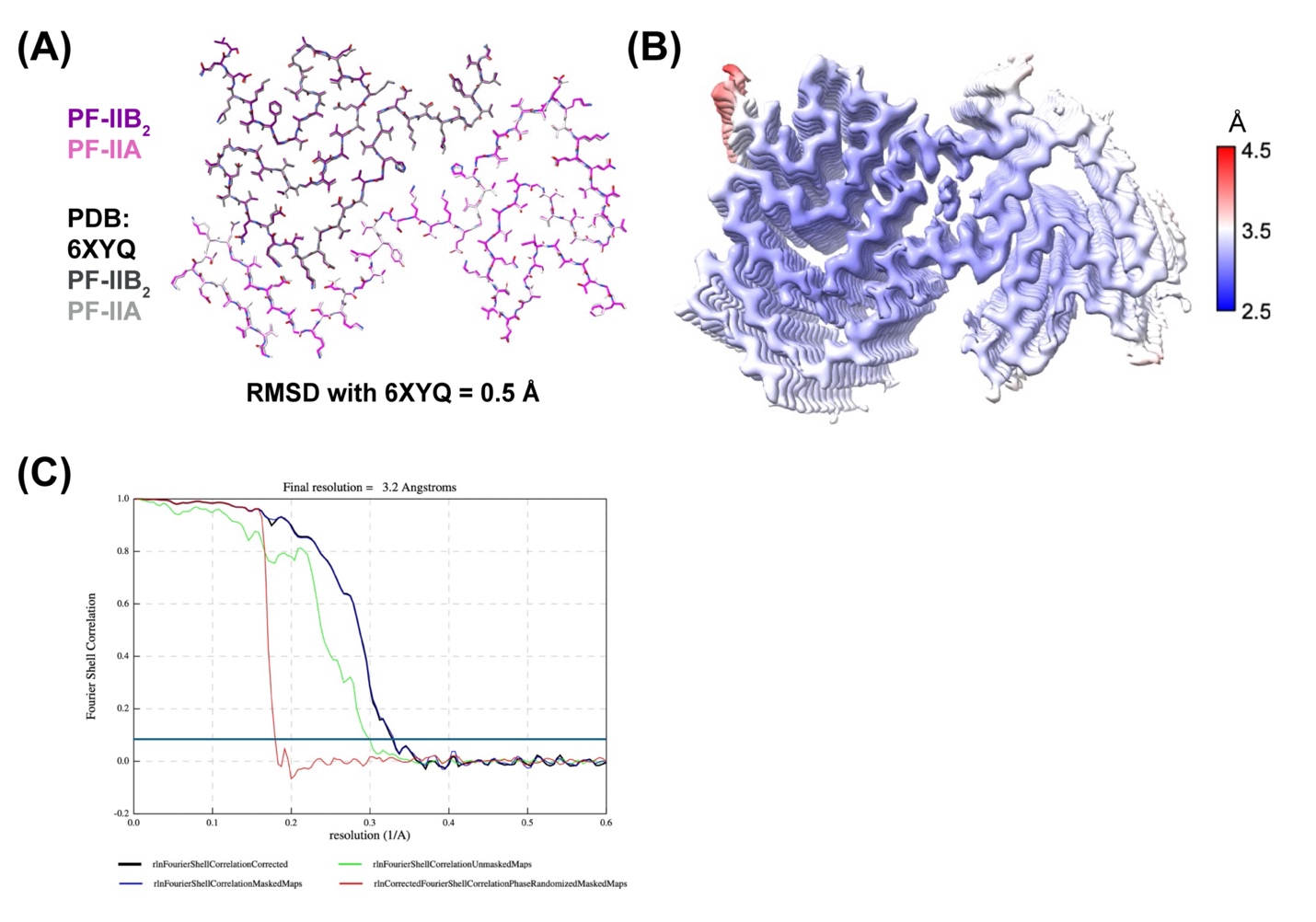


**Fig. S6** Characterization of MSA Type II_2_ filaments in the dataset. (A) Alignment of our Type II_2_ filament model with the previously published model (PDB: 6XYQ). (B) Local resolution of the Type II_2_ filament map. (C) Fourier shell correlation (FSC) curves of the Type II_2_ filament map. The dark blue line denotes the FSC = 0.143 cutoff used for resolution estimation.


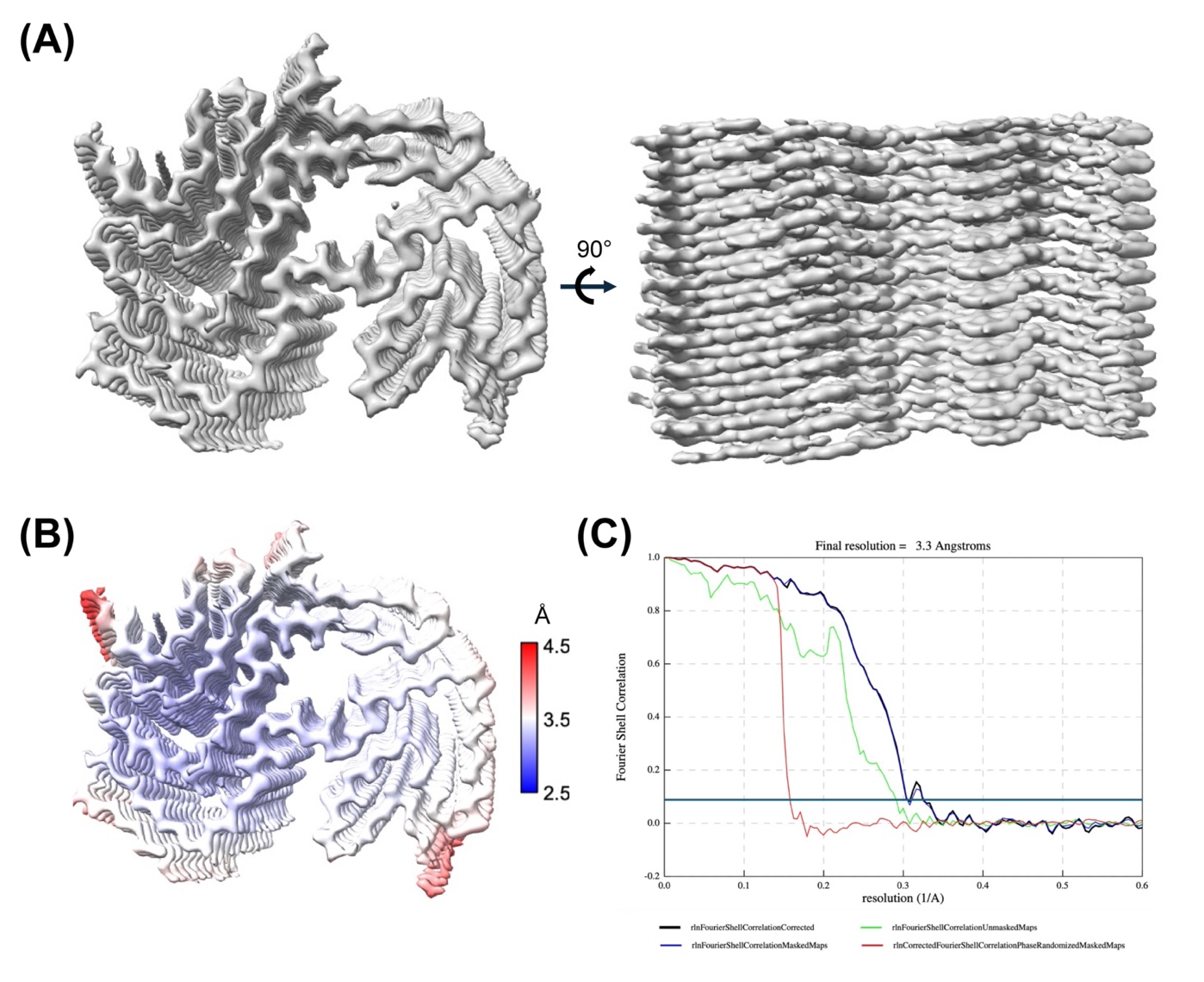


**Fig. S7** Characterization of MSA Type I_2_ filaments in the dataset. (A) Density map of Type I_2_ filaments showing separation between protofilaments in the long axis of the filament. (B) Local resolution of the Type I_2_ filament map. (C) FSC curves of the Type I_2_ filament map. The dark blue line denotes the FSC = 0.143 cutoff used for resolution estimation.

**References**

1. Schweighauser, M., Shi, Y., Tarutani, A., Kametani, F., Murzin, A. G., Ghetti, B., Matsubara, T., Tomita, T., Ando, T., Hasegawa, K., Murayama, S., Yoshida, M., Hasegawa, M., Scheres, S. H. W. & Goedert, M. (2020) Structures of ɑ-synuclein filaments from multiple system atrophy, *Nature.* **585**, 464–469.
